# Monochromatic Phacoemulsification: An Approach for Eyes With Impaired Red Reflex Secondary to Proliferative Diabetic Retinopathy

**DOI:** 10.7759/cureus.105259

**Published:** 2026-03-15

**Authors:** Juan Abel Ramírez-Estudillo, Mauricio Bayram-Suverza, Ximena Ramírez-Galicia, Gustavo Hernandez Ruiz

**Affiliations:** 1 Retina, Fundación Hospital Nuestra Señora de la Luz, Mexico City, MEX; 2 Glaucoma, Asociación para Evitar la Ceguera en México, Mexico City, MEX; 3 Retina, Fundacion Hospital Nuestra Señora de la Luz, Mexico City, MEX

**Keywords:** complex cataract surgery, impaired red reflex, monochrome filter, phacovitrectomy, vitreoretinal surgery

## Abstract

Phacoemulsification requires good visualization of the lens components, which can be challenging during combined vitrectomy. We discuss using the monochrome filter of the NGENUITY® visualization system (Alcon Laboratories, Fort Worth, Texas, United States) for phacoemulsification in complex cases with diminished red reflex, associated with proliferative diabetic retinopathy. Phacoemulsification and 25-gauge vitrectomy were performed by a single surgeon on 12 eyes of 12 consecutive patients with compromised red reflexes, using the 3D digitally assisted visualization system NGENUITY® with a monochrome filter. Following cataract extraction, a standard 25-gauge pars plana vitrectomy was performed. The procedure was completed successfully without complications in all cases. The NGENUITY® monochrome filter enhanced the contrast of ocular structures during continuous capsulorhexis and cortical material removal. This visualization approach was particularly beneficial for phacoemulsification in cases with reduced red reflex due to proliferative diabetic retinopathy, facilitating the procedure and potentially reducing surgical complications like posterior capsule rupture.

## Introduction

Phacoemulsification requires good visualization of the lens components. The visualization of the red reflex using an operating microscope is important for assessing the lens structure and capsule during surgery [1].

In combined vitrectomy procedures, phacoemulsification can be challenging because of the poor fundus reflex caused by factors such as severe vitreous hemorrhage or opacity, corneal edema, and bullous retinal detachment [2].

The surgical risks associated with phacoemulsification in the presence of a poor red reflex include challenges in executing capsulorhexis, an increased likelihood of posterior capsule rupture, and a heightened risk of vitreous loss. Current options to overcome these difficulties include several techniques, mainly involving endoillumination and retroillumination, using various devices to improve the contrast between different parts of the crystalline lens. These techniques offer a suitable solution to the issue of a lack of the red reflex [3-5]. However, these methods have the drawbacks of requiring additional inputs and increased retinal light exposure.

We propose an approach based on the use of a 3D digitally assisted visualization system (3D DAVS) NGENUITY® (Alcon Laboratories, Fort Worth, Texas, United States) along with a monochrome filter as a technique for performing phacoemulsification in complex cases with poor red reflex.

## Materials and methods

Study design

This was a prospective, investigational, nonrandomized, single-arm study conducted at Fundación Hospital Nuestra Señora de la Luz located in Mexico City, Mexico. This study adhered to the guidelines of the Declaration of Helsinki and was approved by the Ethics Committee of the Fundación Hospital Nuestra Señora de la Luz (approval number: 2024R21B2). All patients provided written informed consent.

Inclusion criteria

We included consecutive patients presenting with an impaired red reflex secondary to proliferative diabetic retinopathy and visually significant lens opacities.

Exclusion criteria

Patients exhibiting an abnormal red reflex of origins other than proliferative diabetic retinopathy, such as corneal opacity or vitreous opacities, were excluded from the study. 

Surgical technique

Phacoemulsification and 25-gauge vitrectomy were performed in 12 eyes of 12 consecutive patients with compromised red reflexes. All surgeries were performed by the same surgeon (J.A.R.E.). 

In all cases, the surgeon was positioned in a superior orientation. A 3-mm clear corneal incision was made at the optimal location for each case, as determined by the surgeon. A secondary incision was made when required. Viscoelasticity (Viscoat; Alcon Laboratories, Fort Worth, Texas, United States) was injected into the anterior chamber. The NGENUITY® system monochrome filter was then turned on, and the lights of the operating room were dimmed to enhance the contrast and visualization of the lens components. Continuous curvilinear capsulorhexis was performed using a cystotome and capsular forceps. After cataract extraction, a foldable intraocular lens was implanted. A standard 25-gauge pars plana vitrectomy without a monochrome filter was performed following cataract extraction.

Outcome measures

The primary outcome measure was the occurrence of intraoperative complications during cataract surgery. The surgical time was chronometered and recorded from the surgical videos, considering only the cataract surgery duration. Baseline and three-month postoperative endothelial cell count and best-corrected visual acuity (BCVA) were documented.

Statistical analysis

Analyses were performed using R Version 4.0.5 (R Foundation for Statistical Computing, Vienna, Austria). Continuous variables were presented as mean±standard deviation and median (IQR) as appropriate. Normality of the paired differences (baseline vs. three months) was assessed with the Shapiro-Wilk test. When the paired differences were approximately normally distributed (Shapiro-Wilk p>0.05), a paired Student's t-test was used to compare means, and the mean difference with its 95% confidence interval was reported.

## Results

Cohort and demographics

Twelve eyes of 12 consecutive patients (eight right eyes, four left eyes) undergoing surgery between March 2024 and February 2025 were included. The mean age was 70.3 years (range 55-87); five patients were male, and seven were female. Three patients (25%) exhibited iris neovascularization, and two (16.7%) had posterior synechiae. All eyes had proliferative diabetic retinopathy with markedly reduced red reflex and clinically significant cataract (Table 1).

**Table 1:** Baseline patient characteristics

Patient	Eye	Gender	Age	Red reflex (cause)	Cataract grade	Remarks
1	Right	Woman	68	Impaired (grade II vitreous hemorrhage)	Nuclear 2+, cortical 1+	Iris neovascularization
2	Right	Man	77	Absent (grade IV vitreous hemorrhage)	Nuclear 3+, cortical 2+	Iris neovascularization
3	Left	Woman	62	Impaired (grade III vitreous hemorrhage)	Nuclear 2+, cortical 2+	None
4	Left	Woman	55	Impaired (grade II vitreous hemorrhage + tractional retinal detachment)	Nuclear 1+, posterior subcapsular 3+	None
5	Right	Man	75	Impaired (grade II vitreous hemorrhage + tractional retinal detachment)	Nuclear 1+, posterior subcapsular 2+	Posterior synechiae
6	Right	Woman	87	Absent (grade IV vitreous hemorrhage)	Nuclear 3+	None
7	Left	Man	69	Absent (grade IV vitreous hemorrhage)	Nuclear 2+, posterior subcapsular 2+	Iris neovascularization
8	Right	Woman	63	Impaired (grade III vitreous hemorrhage)	Nuclear 2+, cortical 1+	None
9	Right	Woman	78	Impaired (grade II vitreous hemorrhage)	Nuclear 4+	Posterior synechiae
10	Left	Woman	81	Impaired (tractional retinal detachment)	Nuclear 3+	None
11	Right	Man	57	Absent (grade IV vitreous hemorrhage)	Nuclear 2+, cortical 2+	None
12	Right	Man	71	Impaired (grade II vitreous hemorrhage)	Nuclear 3+, cortical 1+	None

Intraoperative visualization and safety

**Figure 1:**
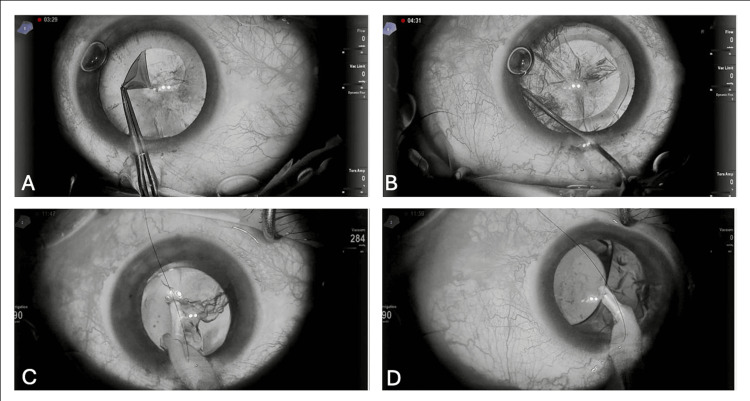
Monochromatic phacoemulsification (A) Continuous circular capsulorhexis is performed using a primary incision. The application of a monochrome filter enhanced the visibility of the anterior capsule, facilitating capsulorhexis. (B) Hydrodissection of the lens cortex. (C) Utilizing a monochrome filter enhances the contrast during lens cortex extraction with the aspiration/irrigation handpiece. (D) Viscoelastic material aspiration.

A combined technique (phacoemulsification with the NGENUITY® monochrome filter followed by 25‑gauge standard vitrectomy) was completed successfully in all 12 cases without conversion to alternative visualization modalities or additional intraocular illumination. The monochrome filter produced a perceptible increase in contrast among the anterior capsule, cortical material, and nucleus, facilitating continuous curvilinear capsulorhexis and cortical removal; this improvement was consistently observed across cases (Figure 1).

There were no posterior capsule ruptures, vitreous loss, or capsule disinsertions. Foldable intraocular lens implantation into the posterior capsule was successful in all 12 eyes.

Surgical parameters and postoperative course

Endothelial cell loss at three months postoperatively was 470.42±154.05 cells, corresponding to approximately 20.4% of the baseline mean (95% CI: 373.44-567.40; p<0.0001). Patients also demonstrated improvement in BCVA: the mean difference between baseline and three months was 1.10±0.41 units, with a 95% CI of 0.84-1.36 (p<0.0001) (Table 2).

**Table 2:** Clinical outcome measures BCVA: best-corrected visual acuity

Variable	Mean±SD	Median (IQR)
Baseline endothelial cell count (cell/mm^2^)	2307.50±283.85	2307.50 (2100-2547.5)
Baseline BCVA (LogMar)	1.83±0.46	1.85 (1.70-2.20)
Surgery time (seconds)	527.83±172.76	460.00 (406-620)
Cumulative dissipated energy	5.14±2.98	5.24 (2.35-6.61)
Endothelial cell count at three months (cell/mm^2^)	1837.08±189.33	1840.00 (1730-1870)
BCVA at three months (LogMar)	0.73±0.25	0.70 (0.65-0.75)

## Discussion

We present an approach employing the NGENUITY® visualization system's monochrome filter as a technique for performing phacoemulsification in complex cases with a diminished red reflex, associated with proliferative diabetic retinopathy. We found that this approach improved the contrast of structures and facilitated the process of cataract extraction. 

Phacoemulsification may be combined with vitrectomy if the cataracts interfere with the surgeon's view of the retina [4]. Phacoemulsification in combined surgery is technically difficult because vitreoretinal alterations can compromise the red reflex necessary to facilitate cataract extraction [1]. Several techniques have been proposed to improve visualization under these circumstances. Lee and Kwok [3] used a multi-port illumination system. However, this system requires special surgical instruments that are unavailable in many countries. Nishimura et al. [5] and Jung et al. [2] used an endoilluminator inside the anterior chamber during phacoemulsification. Yamamoto et al. [4] used trypan blue and endoillumination in the vitreous cavity to enhance visualization during phacoemulsification. Jang et al. [1] used a 23-gauge illuminated infusion chandelier during cataract extraction. All these techniques employ different external lighting systems, successfully enhancing the visualization of lens structures during phacoemulsification with a decreased red reflex. However, the risk of phototoxicity exists when additional lighting devices are used during ophthalmic surgeries, even though the risk may be minimal due to the distance from the retina. This risk can be reduced by using our approach based on the 3D DAVS.

The NGENUITY® is most widely used in vitreoretinal surgery, where it has advantages over conventional microscopic surgery. The 3D DAVS allows surgeons to operate in a more comfortable and physiological "heads‐up" position. It provides a magnified view of the intraoperative field, allowing all personnel in the operating room to see the same image as the surgeon. It has a decreased requirement for endoillumination, reducing retinal phototoxicity [6].

The potential limitations of the NGENUITY® system may include its 0.09-second delay in the image shown on the monitor compared to the image seen directly under the microscope [7]. Another potential negative aspect is the rejection of the learning curve by experienced surgeons who begin operating with this visualization system [8,9].

Compared with vitreoretinal surgery, the manipulation space in cataract surgery is smaller. This lag is more evident in anterior segment maneuvers, because surgical manipulations in the anterior chamber are usually faster than those in the posterior chamber [7].

Several studies have shown a lack of significant differences in the surgical time and loss of endothelial cells with the use of 3D DAVS compared to a conventional microscope in anterior segment surgeries [8,9].

Although the major concern regarding the use of the 3D DAVS system in anterior segment surgeries reported in the literature is the time lag, the current system has a <70-ms time lag. The human brain does not recognize time lags ≤50 ms. Moreover, this time lag is only appreciated during the learning phase, while in the post-learning phase, surgeons do not perceive a time lag, and it does not impact surgical outcomes [8].

The optical benefits of 3D systems include an expanded field of view, improved depth perception, enhanced color contrast, and more efficient illumination. The 3D DAVS system ensures a wide, high-resolution field of view, even at high levels of magnification, while maintaining resolution throughout the entire display [8]. This system also maintains a consistent depth of field at high magnifications, ensuring clarity across all layers of focus, and provides finer depth resolution at the highest system magnification [8,9]. Increased magnification amplifies the view for intricate tasks, finer resolution helps maintain focus across an expanded surgical space, and increased depth resolution aids in resolving fine details [9].

Additionally, digital modulation in a 3D DAVS system allows for better imaging, enabling surgeons to operate at lower illumination levels. In fact, the illumination required in a 3D system is only 1/10th of that required with a traditional microscope [9,10]. A 3D DAVS camera is extremely sensitive to light, which allows it to capture images using a reduced camera aperture. A decrease in the camera aperture enhances the depth of field; however, it also reduces the light reaching the camera's sensor, making the images appear darker. Despite reducing the camera aperture to 30%, the depth of field with NGENUITY® was two to three times greater than that obtained using a conventional microscope [8,9].

In addition to the well-known advantages of the NGENUITY® system, which allows for excellent visualization of the lens structure, thickness, and depth, the use of a monochrome filter enhances contrast and facilitates the visualization of the lens capsule and other components. This may reduce the risk of intraoperative complications, such as posterior capsule rupture during phacoemulsification procedures in cases where the red reflex is lacking. Furthermore, compared with other previously published approaches for phacoemulsification with a compromised red reflex, our technique eliminates the need for additional illumination devices, reduces costs, and minimizes the risk of retinal phototoxicity [7].

The patients in this study experienced a clinically significant mean endothelial cell loss; however, this finding is consistent with previously published reports. Diabetic patients exhibit detectable corneal endothelial alterations, including reduced baseline endothelial cell density and morphological changes, such as increased cell size variability and decreased hexagonality, indicative of diminished endothelial reserve [11]. Meta‑analytic evidence supports a modest but consistent reduction in endothelial cell density and greater dysmorphism of endothelial cells in diabetics versus non‑diabetic controls [11,12]. These baseline abnormalities predispose diabetic corneas to greater susceptibility to intraoperative injury; comparative studies report increased postoperative endothelial cell loss in diabetic cohorts [12,13]. Pathophysiologic contributors include chronic hyperglycemia-induced metabolic and oxidative stress, accumulation of advanced glycation end products, impaired Na+/K+‑ATPase activity with reduced pump function, and an amplified or prolonged postoperative inflammatory response, all of which may impair endothelial recovery [11,13].

The improvement in BCVA reflects the combined therapeutic effects of cataract extraction and the concomitant vitreoretinal procedures performed during the surgery. Therefore, while the monochrome may facilitate intraoperative visualization and technically assist the procedures, causal attribution of functional outcomes to the visualization modality alone is not supported by our study.

One limitation of this approach is the limited availability of the NGENUITY® system in operating rooms. However, we believe it to be a viable alternative for patients with a poor red reflex requiring phacoemulsification combined with vitrectomy, in settings where this technology is accessible.

Limitations of the study include the small sample size and the lack of a comparison group. Therefore, larger comparative studies with robust controlled designs are needed to confirm the benefits and quantify effect sizes.

## Conclusions

Employing the NGENUITY® monochrome filter for phacoemulsification in challenging cataract cases with reduced red reflex secondary to proliferative diabetic retinopathy was particularly beneficial during continuous capsulorhexis and cortical material extraction. This visualization approach facilitates the procedure and may help decrease surgical complications, such as posterior capsule rupture.

In this study, we demonstrate that in operating rooms equipped with this technology, the use of a monochrome filter is a safe and effective approach to facilitate cataract surgery in eyes with a poor red reflex, obviating the need for additional illumination sources. It is important to emphasize that the findings of this study are preliminary and require confirmation in larger controlled studies.
